# Distractor displacements during saccades are reflected in the time-course of saccade curvature

**DOI:** 10.1038/s41598-018-20578-9

**Published:** 2018-02-06

**Authors:** Jonathan van Leeuwen, Artem V. Belopolsky

**Affiliations:** Department of Experimental and Applied Psychology, Vrije Universiteit, Amsterdam, The Netherlands

## Abstract

Every time we make a saccade we form a prediction about where objects are going to be when the eye lands. This is crucial since the oculomotor system is retinotopically organized and every saccade drastically changes the projection of objects on the retina. We investigated how quickly the oculomotor system accommodates new spatial information when a distractor is displaced during a saccade. Participants performed sequences of horizontal and vertical saccades and oculomotor competition was induced by presenting a task-irrelevant distractor before the first saccade. On half of the trials the distractor remained in the same location after the first saccade and on the other half the distractor moved during the first saccade. Curvature of the second saccade was used to track target-distractor competition. At short intersaccadic intervals, saccades curved away from the original distractor location, confirming that in the oculomotor system spatiotopic representations emerge rapidly and automatically. Approximately 190 ms after the first saccade, second saccades curved away from the new distractor location. These results show that after a saccade the oculomotor system is initially driven by the spatial prediction made before the saccade, but it is able to quickly update these spatial predictions based on new visual information.

## Introduction

Every time we make a saccade we form a prediction about where objects are going to be when the eye lands. This is crucial since the oculomotor system is retinotopically organized (eye-centered) and every saccade drastically changes the projection of objects on the retina. As a result of these predictions, the visual world is perceived as stable and we do not feel disoriented, which allows us to perform complex actions. Despite this remarkable ability to act in the presence of intermittent visual input, humans are notoriously bad in detecting changes to positions of objects across saccades^1–4^. This shows a discrepancy between representations in the motor system and conscious experience of visual stability^5,6^.

The problem of visual stability has preoccupied the minds of researchers for several centuries (for a review see^6^). The main accounts of visual stability can be roughly divided in two categories. The first group of theories, dating back to Helmoltz^7,8^, emphasized the role of so-called extra-retinal signals in achieving visual stability. One such signal is a copy of the oculomotor program (the efference copy), which was thought to be used for correction or cancellation of the displacement of retinal image induced by the eye movement (i.e. the cancellation theories). For example, Helmholtz already noted that when patients with eye muscle paralysis attempted to move their eyes they experienced a shift in the retinal image, although objectively the image remained stationary on the retina^8^. Consistent with this idea it has been demonstrated that receptive fields of neurons in lateral intraparietal area (LIP) shift to compensate for saccade about 80 ms before the start of the eye movement^9,10^ (for a review see^11^). This predictive remapping of the receptive fields before saccades has also been observed in other brain areas involved in oculomotor control, such as frontal eye fields (FEF) and superior colliculus (SC,^12–14^).

The cancellation theories cannot explain, however, the fact that observers tend to miss object displacements if they occur during a saccade^15^. The information about the target location is not lost, since when target is not continuously present before and after the saccade, but reappears at a displaced location after a short blank interval (50 ms) after the saccade, the displacements are detected with high accuracy^1–3^. This “target blanking” shows that the prediction about target location based on the extra-retinal signal is highly accurate, but is ignored. Instead, the visual system assumes as a null hypothesis that the visual world is stable across saccades (i.e. the assumption theories). A large discrepancy between the predicted and actual target location violates this assumption and allows the target displacement to be detected. Importantly, target blanking also violates this assumption since in this case the pre-saccadic object is not immediately available after the saccade.

Recent computational models have provided insight into possible mechanisms of saccadic suppression of displacement. One influential model proposed that pre- and post-saccadic information about saccade target location is integrated in a statistically optimal fashion^16^. This means that perception is driven by a combination of relative reliability of the information available before and after saccade. Therefore, the perception of stability of a saccade target is shifted towards the post-saccadic target. The model predicts that the threshold for detecting displacements can be biased by noise, such as individual variation in saccade landing positions^16^. A similar model explains why large displacements are detected better than small displacements by positing that the pre- and post-saccadic percepts are only integrated if the discrepancy between the two signals is small. If the discrepancy is large, integration does not occur and the assumption of object constancy is discarded^17^. While previous models were able to explain saccadic suppression of displacement, but not the effect of target blanking, this was achieved in a recent computational model which took into account the neurophysiology of the visual system^18,19^. This model posits that under normal viewing circumstances pre- and post-saccadic information are recurrently integrated. If targets are blanked during saccades the pre-saccadic information becomes unreliable and the percept gets weighted towards the post-saccadic information. It is important to note that saccadic suppression of displacement is measured by a subjective report of whether object displacement was consciously perceived.

It seems that while conscious perception is easily fooled by target displacements, the oculomotor system knows exactly what is going on. First of all, when a target is displaced during a saccade, the eyes make a “corrective” eye movement to the new target location, but this still does not lead to perception of target displacement^1^. Second, even though the target displacements are not consciously perceived, the oculomotor system quickly adapts to these displacements by increasing or decreasing the saccade amplitude on the following trials accordingly (i.e. saccade adaptation^20^). It appears as if the oculomotor system contains accurate information about space^21,22^ but that the information does not necessarily reach conscious awareness. Therefore, in order to understand how spatial representations in the oculomotor system are updated across eye movements, it is imperative to use implicit measures instead of relying on subjective reports.

The fact that the oculomotor system rapidly updates information about objects across saccades has recently been demonstrated by Jonikaitis & Belopolsky^23^. They used saccadic curvature to examine whether the competition between target and distractors across eye movements occurs in retinotopic or spatiotopic coordinates. Saccade curvature has been attributed to competition in the oculomotor spatial priority map for potential saccade targets in the intermediate layers of superior colliculus^24^. Strong target-distractor competition has been attributed to saccade curvature towards distractor locations, whereas successfully resolved competition has been suggested to result in curvature away from the distractor location^25^. In the study by Jonikaitis & Belopolsky^23^ participants performed a sequence consisting of a horizontal and a vertical saccade and the oculomotor competition was induced by briefly presenting a task-irrelevant distractor at different times during the sequence. Despite the intervening saccade, the second saccade curved away from a spatial representation of the distractor that was presented before the first saccade, suggesting that in the oculomotor system spatiotopic representations emerge rapidly and automatically. Saccade curvature provided an excellent implicit measure of oculomotor updating without the need for a dual-task or subjective report.

The goal of the present study was to investigate how quickly the oculomotor system accommodates new competing visual information. Measuring saccade curvature allowed us to precisely track how target-distractor competition in the oculomotor system was updated over time when a distractor changed location during a saccade, without interfering with the updating process. To examine this, we modeled our paradigm after Jonikaitis & Belopolsky^23^, but presented the distractor continuously across the first saccade. Importantly, on half of the trials the location of the distractor was displaced during the first saccade. The trajectory of the second saccade was used to assess whether the original (before first saccade) or new (after first saccade) distractor location was represented in the oculomotor system. Crucially, we took advantage of the variability in the time interval between the two saccades to determine when the original distractor representation switched to the new distractor representation.

## Experiment 1

In Experiment 1 participants were instructed to make sequences consisting of a horizontal and a vertical saccade while ignoring a distractor occurring slightly before the start of the sequence. On half of the trials the distractor remained at the same spatial location during the whole saccade sequence. On the other half of the trials distractor was displaced during the first saccade. If the oculomotor system accurately represents spatial locations across saccades the second saccade is expected to curve away from the original distractor location when the intersaccadic interval is short (as in^23^). At some point in time the new distractor representation is expected to become dominant in the oculomotor system and the second saccade would start to curve away from the new location. It is also possible that the new distractor representation captures attention and rapidly overrides the representation in the oculomotor system, similar to what happens during saccadic suppression of displacement. In that case, the eyes should curve away from the new distractor location already at the shortest intersaccadic intervals.

### Methods

#### Setup and calibration

The experiment was conducted in a dimly lit room. The stimuli were presented on a 21” LCD monitor (Samsung 2233RZ) with a resolution of 1680 × 1050 with refresh rate of 120 Hz. Participants viewed the screen from a distance of 75 cm. A chin and forehead rest ensured that the correct head position was maintained during the experiment. Left eye gaze position was recorded with the Eyelink 1000 (SR Research), sampling at 1000 Hz. A nine-point automatic calibration was used and repeated until the max validation offset was less than 1° and the average validation offset was less than 0.5°. The calibration and experiment backgrounds were always gray (40 cd/m^2^). The calibration and experiment target dots were always black (0 cd/m^2^). A beep was played after each correct fixation of a calibration dot. The participant was alone in the room during the calibration.

#### Participants

Fourteen naïve participants took part in Experiment 1. Three out of the fourteen participants were either unable to pass the screening (see stimuli, design and procedure) or were unable to maintain a stable calibration. Eleven naïve participants, four females (mean age: 22) and seven males (mean age: 26) were included in the experiment. Participants received either money (8€ per hour) or credits (60 credits per hour) as compensation for their time. All participants had normal or corrected to normal vision, the details of the experiment were explained and they gave their informed consent prior to participating. Both Experiment 1 and 2 were conducted with approval of the local ethics committee of the Vrije Universiteit Amsterdam and all rules, regulations and guidelines were followed.

#### Stimuli, design and procedure

**Figure 1:**
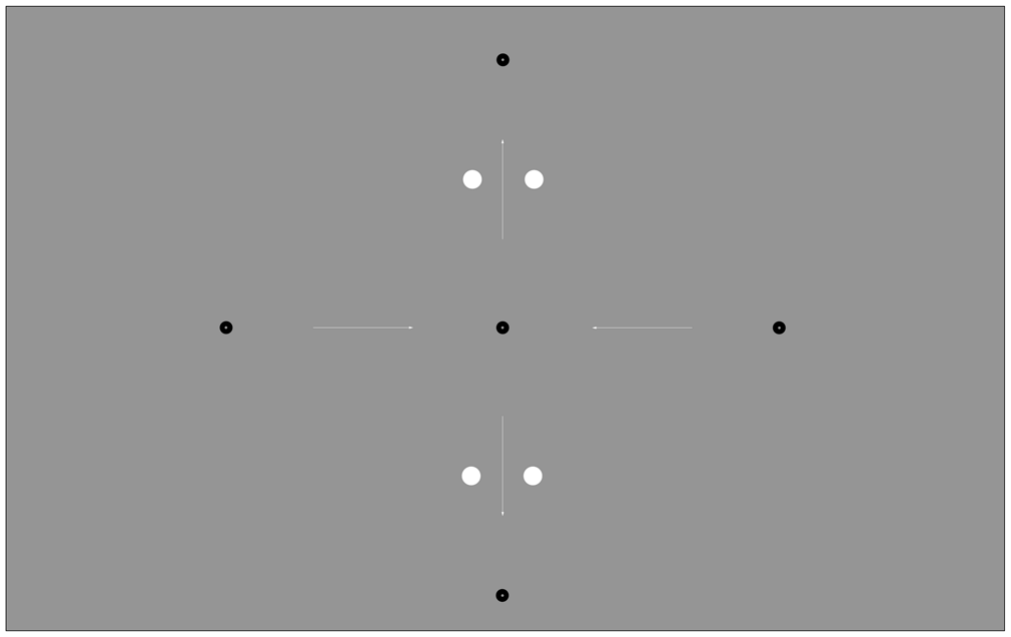
The four possible saccade directions and all distractor positions used in Experiments 1 and 2. Participants always made the first saccade to the center dot from either the left or the right side, then the second saccade up or down. Black dots are fixation/saccade targets, white dots are distractors dots, and white arrows illustrate possible saccade directions. The dot sizes and positions are drawn to scale. White arrows are shown for illustration purposes only. Distractor dots were only displaced horizontally and only one distractor was displayed per trial.

**Figure 2:**
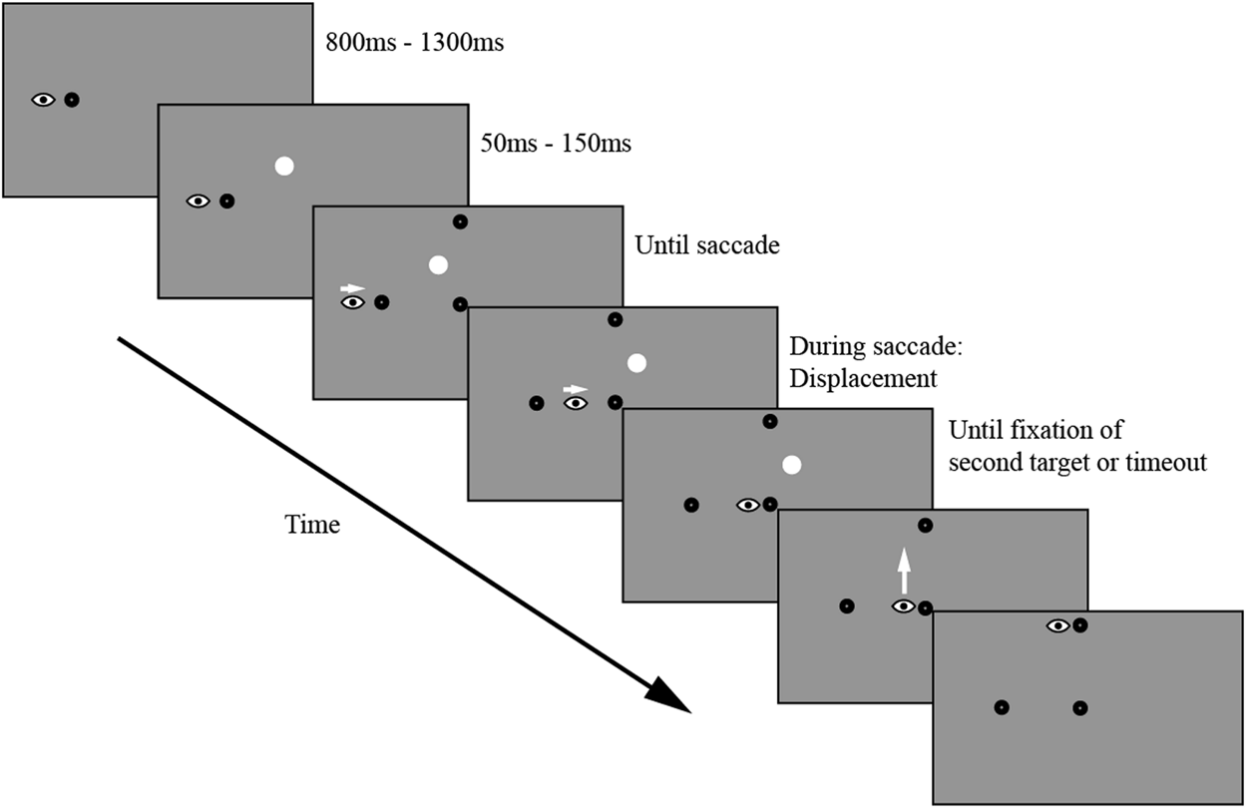
An example trial showing the displacement condition in Experiments 1 and 2. In the no displacement condition the distractor stayed at the same location throughout the whole saccade sequence. In Experiment 2 a post-saccadic condition was introduced, which was similar to the displacement condition, except that no distractor was present before the saccade sequence started, but was presented during the first saccade until the end of the trial. The eye represents the current gaze position and the white arrows indicate saccade directions. It should be noted that the figure is not to scale.

The stimuli consisted of one fixation dot, two saccade target dots and a distractor dot. The fixation and target dots were black, had a radius of 0.25° and a central aperture with a radius of 0.05° to facilitate fixation. The fixation dot was presented 10° to the left or right of the screen center. The first saccade target was presented at a random horizontal position between 10° and 12° degrees towards the center of the screen relative to the fixation dot. The second saccade target was presented randomly between 9° and 10° above or below the first saccade target (Fig. 1).The distractor was a white dot (122 cd/m^2^) with a radius of 0.38°. The vertical distance between the distractor dot and the first saccade target dot varied randomly between 5° and 6°. The horizontal distance between the distractor dot and the first saccade target was ± 0.75°. In the displacement condition the distractor dot was displaced 1.5° during the saccade, crossing the imaginary straight line between the saccade targets (see Fig. 2).

We used a 3(distractor displacement: no displacement vs. displacement) × 4(saccade direction) × 2(distractor position: clockwise vs. counter-clockwise, relative to the second saccade target) design, manipulated orthogonally within-subjects in each block.

The participants were instructed to make a sequence of a horizontal and vertical saccades (see Fig. 2). They were told that a white distractor dot would appear and were instructed to ignore it. In each session the participants first performed a practice block followed by 18 experimental blocks. All blocks were identical and consisted of 64 trials. Each trial started with participants fixating the dot on either the left or the right side of the screen. The trial started when fixation was detected (Fig. 2). The start of fixation was defined as the first gaze sample in a trial which was less than 3° of Euclidian distance from the fixation dot. After fixating the dot for a random time interval (800–1300 ms) a white distractor dot was presented. 50–150 ms after the presentation of the distractor the first and second saccade target dots were presented simultaneously. The participants were instructed to keep fixating the fixation dot until the target dots were presented, then to look at the first and second saccade targets as fast and accurate as possible. The distractor displacement was triggered if the distance between gaze position and fixation dot was greater than 2° and the distance between gaze position and the first saccade target was less than 2°. This was done to ensure that the displacement only happened during a saccade towards the first target. If the first saccade was directed towards the distractor at the moment the distractor dot was displaced, the trial was aborted and the distractor dot was removed. The trial ended when gaze was within 3° of the second saccade target or until trial timeout (1500 ms after onset of saccade targets). The no displacement condition was identical to the displacement condition with the exception that the distractor stayed at the same location during the trial.

A saccade was considered correct if the gaze position at any time after target onset was within 3° of the target dot. If the trial was too slow or one of the saccades was incorrect the trial was logged as incorrect. After each trial participants received feedback about saccade accuracy, e.g., if they made correct saccades the saccade targets turned green (79 cd/m^2^), if they were incorrect they turned red (31 cd/m^2^). If the second saccade landed more than 450 ms (the screening task) or 550 ms (the experimental task) after the onset of the saccade targets an auditory feedback sound was played immediately after the saccade landing. The feedback sound was a sine wave at 800 Hz with a decay time of 5 ms and duration of 100 ms. After each block participants received feedback on their performance (accuracy and saccade latency) in the preceding block. If the number of correct trials was lower than 80% or the average latency (time from the target onset until correct fixation of the second target) of the correct trials was slower than 500 ms, they were prompted to do better.

The experiment consisted of two sessions of approximately 1.5 hours each. At the start of the first session participants had to perform a screening task. The screening task was the same as the experimental task but without the distractor dot. The screening task consisted of 5 blocks of 32 trials. If the participants completed the sequence of saccades with accuracy greater than 90% and with the average correct saccade latency shorter than 450 ms, the screening task was stopped and they could continue with the experimental task. Three of the fourteen participants were unable to fulfill these criteria and were not allowed to continue with the experiment.

#### Data pre-processing

Saccades where defined using an acceleration threshold of 9500°/s^2^, a velocity threshold of 35°/s and were automatically detected by the Eyelink system. Using custom code, all relevant events and data were extracted for each trial. The first saccade was defined as the first saccade which started after the targets were displayed. The second saccade was defined as the first saccade which started from the first target and ended at the second target (any corrective saccades after the first saccade were ignored if they started and ended within 3° of the first target dot).

**Figure 3:**
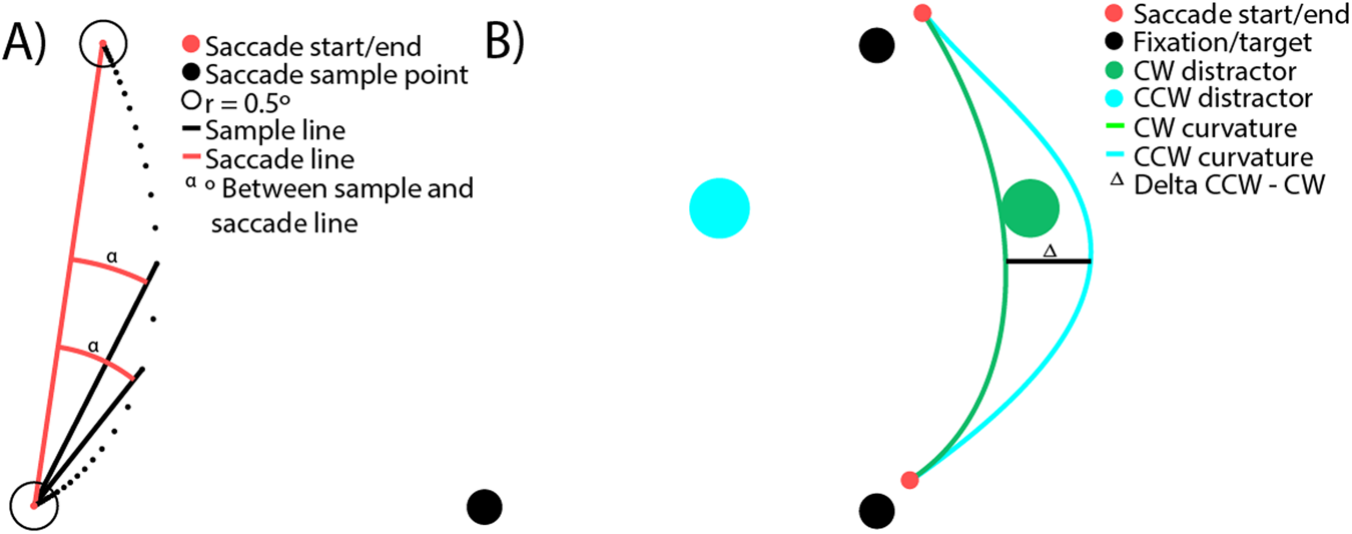
(**A**) Illustration of saccade curvature calculation. The curvature is the median angle α of all angles between each sample point and the straight line between the start and end point of the saccade (red line) which are further away from the start and endpoints than 0.5° of Euclidian distance (black circle around the start and end points). (**B**) Calculating curvature difference, relative to CW and CCW distractors. The single value curvature difference for the conditions is the difference between the curvature in the CW and CCW trials.

The data from different saccade sequences was transformed to fit into the same reference frame (first saccade from left, second saccade upwards) before collapsing the data. After rotation the distractor could either be in the clockwise (CW) position or in the counter-clockwise (CCW) position relative to the second target (see Fig. 3B). All rotations were made relative to the initial distractor location.

Twelve criteria for rejecting trials were used. Combined over all included participants there were a total of 25,344 trials in the experiment of which a total of 35.3% were rejected (see Supplementary Table 1 for a detailed list of rejection criteria and the percentage of trials rejected for each criterion). Due to the stringent exclusion criteria, only one scan path was included for analysis. The scan path consisted of initial fixation followed by a saccade to the first target, followed in turn by a saccade to the second target. This resulted in inclusion of trials with one or more corrective saccades after landing on the first target. Specifically, 90.6% of the included trials in Experiment 1 had 0 corrective saccades, 9.3% had 1 corrective saccade and 0.1% had 2 corrective saccades.

#### Saccade curvature calculation

Saccade curvature was calculated as the median angle between each sample point in a saccade and an imaginary line between the start and end point of the saccade (Fig. 3A). All points within 0.5° of the saccade start/endpoint were discarded before calculating saccade curvature. The curvature values were split into four conditions; no displacement CCW, no displacement CW, displacement CCW and displacement CW. Curvature difference was defined as the difference between the curvature on the trials where the distractor was presented in the CW and CCW positions, separately for no displacement and displacement trials. A positive curvature difference reflects curvature away from the distractor and negative curvature difference reflects curvature towards the distractor (see Fig. 3B for an example of curvature away from distractor). It is important to note that curvature calculations were done referenced to the location of the distractor at the start of saccade sequence. This means that positive curvature difference in the no displacement condition reflects curvature away from the distractor. For the displaced condition a positive curvature difference reflects curvature away from the original (before displacement) location and negative curvature difference reflects curvature away from the displaced position. In the post-saccade condition in Experiment 2, positive curvature difference reflects curvature towards the distractor and a negative curvature difference reflects curvature away from the distractor. Also note that all statistics and plots are done using the curvature difference.

#### Gaussian smoothing

To get a precise estimate of the time-course of saccade curvature as a function of intersaccadic interval, we smoothed the curvature using a moving Gaussian window between 100 and 300 ms (step size 1 ms and σ = 8 ms). A smoothed curvature time series was made for each of the four conditions for each individual subject before calculating curvature difference for each time-point (see curvature calculation).

#### Cluster-based permutation testing between conditions

To determine when there was significant curvature differences between the two conditions (no displacement, displacement) a weighted within-subjects t-test was done for each time point of the Gaussian smoothed data. Clusters of significant differences were defined as two or more consecutive time points with p < 0.05, and the size of each cluster was defined as the sum of t-values in the cluster. To control for multiple comparisons, cluster-based permutation testing was used to determine clusters’ significance. The curvature data was divided into CW and CCW trials, randomly assigned to either the no displacement or displacement condition, smoothed and curvature difference was calculated. This was done ten thousand times for each participant and the sum of t-values for the largest cluster size in each permutation was used to build the permutation distribution. The p-value of the clusters found in the non-permutated data is the proportion of clusters in the permutation distribution with equal or larger sum of t-values than the clusters in non-permutated data.

#### Software

The experiment was programmed using OpenSesame^26^. Data processing was done with Python^27^ and statistics were done with IBM SPSS^28^. Figures were made using Adobe Illustrator (CS5.1).

### Results

**Figure 4:**
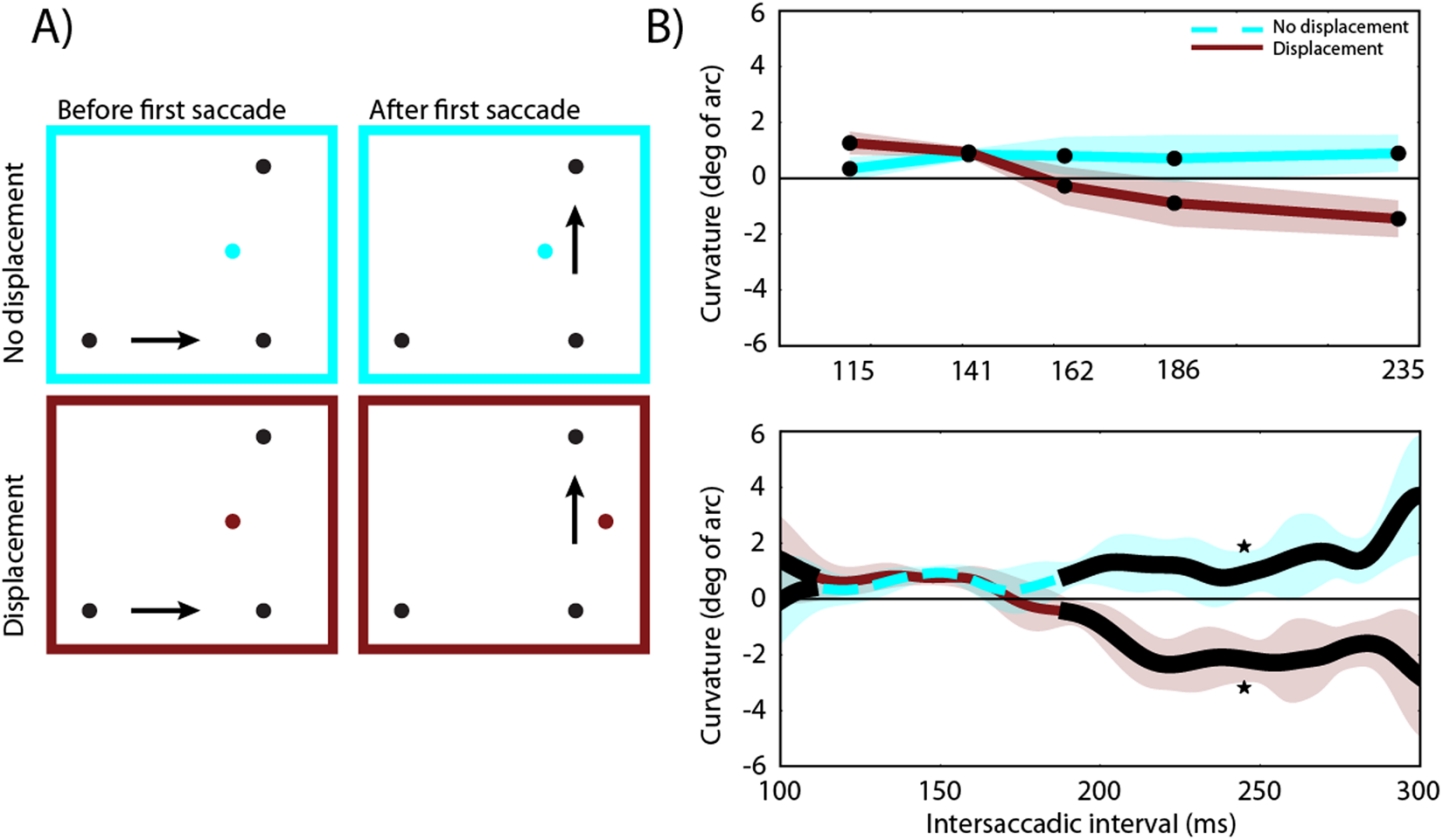
The results of Experiment 1. (**A**) The position of the distractor before the first saccade and the position of the distractor after the first saccade for each condition. (**B**) Amount of curvature difference (degrees of arc) as a function of inter-saccadic interval for each condition. Upper panel: Quintile binned data. Lower panel: Data smoothed with a Gaussian kernel, with the black line indicating time points were a weighted within-subjects t-test resulted in *p* < 0.05 between conditions. In the no displacement condition positive values indicate curvature away from the distractor. In the displacement condition positive values indicate curvature away from the pre-displacement distractor. The shaded areas are 95% within-subjects confidence intervals. Asterisks indicate significant cluster(s).

The time-course of saccade curvature in distractor displacement and no displacement conditions is plotted in Fig. 4. To examine the time-course of updating the distractor position across saccades, curvatures for each condition and distractor position were split into quintiles based on the inter-saccadic interval. Then curvature differences were calculated for each bin (see *Saccade curvature calculation* section). A two-factor repeated-measures ANOVA with Condition (no displacement, displacement) and time (bins 1–5) was conducted on saccade curvature. The assumption of sphericity was rejected as the Mauchly’s test of sphericity was significant for time, *Mauchly’s W*(9) = 0.033, *p* < 0.001, and Greenhouse-Geisser correction was applied. There was no significant main effect of condition, *F*(1, 10) = 4.02, *p* = 0.073. There was a significant main effect of Time, *F*(2.032, 20.32) = 4.43, *p* = 0.025, as well as a significant Condition x Time interaction (*F*(2.9, 29.5) = 8.75, *p* < 0.001. Indicating that the time-course of saccade curvature differed between conditions (Fig. 4B, upper panel).

Post-hoc comparisons for each time bin showed a significant effect between conditions at bin 5,*t*(10) = 3.8, *p* = 0.015, with both conditions showing curvature away from the post-saccadic distractor location. The reported p-value is Bonferroni-corrected. Based on bins, the statistics suggest that it takes the oculomotor system about 235 ms to switch from the old distractor location to the new distractor location.

Since temporal precision is lost during binning, a novel method was used to improve the temporal precision of the signal. Curvature differences as a function of time were extracted by smoothing curvature over time using a Gaussian kernel, which does not limit the temporal precision to an arbitrary number of bins and reflects the entire time-course (see Methods and Fig. 4). The smoothed time-course showed a similar pattern of curvature over time as binning, but allowed more precise estimation of when curvature started to differ between the conditions.

Within-subjects t-tests and cluster-based permutation testing was used to determine when the two conditions differed significantly. The results show that there is a small, but non-significant (*p* = 0.5), cluster at the shortest times while there is a large significant cluster starting at about 180 ms (*p* < 0.001) which stays significant throughout the remaining time-window. This shows that curvature in the two conditions follow the same pattern over time until approximately 180 ms, at which point curvature away from the original distractor location in the displacement condition switches to curvature away from the displaced (new) distractor location.

### Discussion

The results from Experiment 1 clearly show that saccades curve away from pre-saccadic spatiotopic locations at short inter-saccadic intervals. Indicating that the oculomotor system rapidly updates task-irrelevant visual information to a spatiotopic reference frame, replicating previous findings^23^ and falling in line with research on rapid spatiotopic updating^22,29–31^. Interestingly, in the displacement condition curvature switched from being away from the predicted spatiotopic location to being away from the new displaced location after 180 ms. These results suggest that the oculomotor system initially relies on predictions made before the initial saccade, but that these predictions can be updated after approximately 180 ms.

## Experiment 2

Experiment 1 clearly showed that when the distractor was displaced the eyes curved away from the original distractor location early in time, while later in time the eyes curved away from the new distractor location. Experiment 2, was done to replicate and extend these results by ruling out an alternative explanation. It is known that short-latency saccades (less than 200 ms) tend to curve towards the distractor, while saccades with a longer latencies curve away from the distractor^25^. Within that framework, the early curvature away from the original distractor location followed by curvature away from the new distractor location, observed in Experiment 1, could also be interpreted as curvature towards the new distractor location, replaced by curvature away from the new distractor location later in time. Note that this explanation is already inconsistent with the results of Jonikaitis & Belopolsky^23^, who observed early curvature away from the original distractor location.

To rule out the alternative explanation a new condition was added in Experiment 2. In this post-saccadic distractor condition, there was no distractor present at the start of the saccade sequence, but instead, the distractor was presented during the first saccade. If in Experiment 1 the eyes first curved towards and then away from the new distractor location, then such a time-course should also be observed in the post-saccadic condition. If, however, our original interpretation was correct, then there should be no curvature towards the distractor in the post-saccadic condition, but instead curvature away from the distractor should already be evident at the shortest inter-saccadic intervals.

### Methods

#### Participants

Twenty naïve participants took part in Experiment 2. Six out of the 20 participants were either unable to pass the screening (described in Methods of Experiment 1) or were unable to maintain a stable calibration. Participants were also excluded if the amount of data rejected was more than the average rejection percentage across all participants plus one standard deviation. This resulted in four additional participants being excluded from the data analysis. Therefore, ten naïve participants, four females (mean age: 23) and six males (mean age: 27) were included in Experiment 2. Participants received either money (8€ per hour) or credits (60 credits per hour) as compensation for their time. All participants had normal or corrected to normal vision, the details of the experiment were explained and they gave their informed consent prior to participating. The experiment was conducted with approval of the local ethics committee of the Vrije Universiteit Amsterdam.

#### Stimuli, design and procedure

The stimuli were exactly the same as in Experiment 1. The design was changed to a 3 (distractor: no displacement vs. displacement vs. post-saccadic onset) x 4 (saccade direction)x 2 (distractor position: clockwise vs. counter-clockwise) design, manipulated orthogonally within-subjects in each block. The difference in the post-saccadic distractor condition was that the distractor was absent until the first saccade and presented during the first saccade.

#### Data pre-processing

The data pre-processing steps were the same as in Experiment 1. Combined over all included participants there were a total of 23,040 trials of which a total of 46.5% were rejected (see Supplementary Table 1 for a detailed list of rejection criteria and the percentage of trials rejected for each criterion). Due to the stringent exclusion criteria, only one scan path was included for analysis. The scan path consisted of initial fixation followed by a saccade to the first target, followed in turn by a saccade to the second target. This resulted in inclusion of trials with one or more corrective saccades after landing on the first target. Specifically, 89% of the included trials in Experiment 1 had 0 corrective saccades, 10.9% had 1 corrective saccade and 0.1% had 2 corrective saccades.

### Results

**Figure 5:**
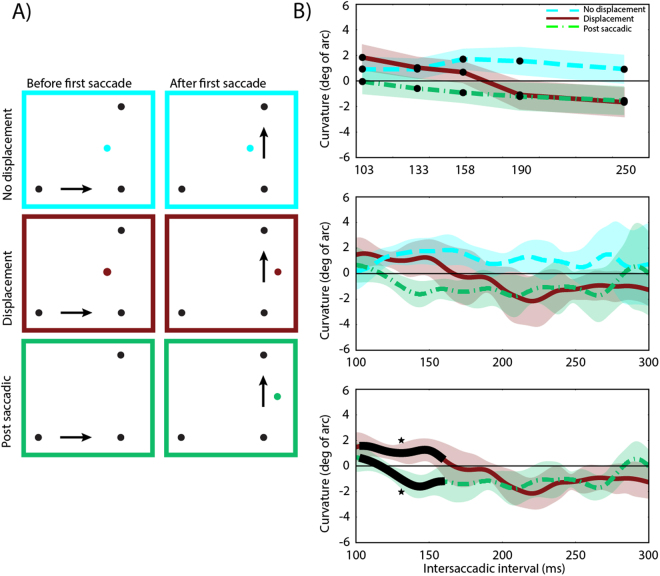
The results of Experiment 2. (**A**) The position of the distractor before the first saccade and the position of the distractor after the first saccade for each condition. (**B**) Amount of curvature difference (degrees of arc) as a function of inter-saccadic interval for each condition. Upper panel: Quintile-binned data. Middle panel: Data from the three conditions smoothed with a Gaussian kernel. Lower panel: Data smoothed with a Gaussian kernel, with the black line indicating time points were a weighted within-subjects t-test resulted in *p* < 0.05 between the displacement and post-saccadic conditions. In the no displacement condition positive values indicate curvature away from the distractor. In the displacement condition positive values indicate curvature away from the pre-displacement distractor. In the post-saccadic condition negative curvature reflects curvature away from the distractor. The shaded areas are 95% within-subjects confidence intervals. Asterisks indicate significant cluster(s).

The time-courses of saccade curvature in distractor displacement, no distractor displacement and post-saccadic distractor conditions are plotted in Fig. 5. Similar to Experiment 1 we first split the data into quintiles based on time and calculated curvature away for each condition. A two-factor repeated-measures ANOVA with Condition (no displacement, displacement, post saccade) and time (bins 1–5) was conducted on saccade curvature. The assumption of sphericity was rejected as the Mauchly’s test of sphericity was significant for time and the interaction, *Mauchly’s W*(9) = 0.076, *p* < 0.028, *Mauchly’s W*(35) < 0.001, *p* < 0.009, respectively, the F-values reported for time and interaction are therefore Greenhouse-Geisser values. There was a significant main effect of condition, *F*(2, 18) = 8.213, *p* = 0.003, a significant main effect of time, *F*(2.261, 20.351) = 6.073, *p* = 0.007, as well as a significant interaction between condition and time, *F*(2.767, 76.055) = 6.358, *p* < 0.003. This indicates that saccade curvature depended on condition, time and the interaction between condition and time (Fig. 5B upper panel).

The same post-hoc comparisons as in Experiment 1 were done between the no displacement and displacement conditions. The results from Experiment 1 were replicated with a significant effect between conditions at bin 5, *t*(9) = 3,602, *p* = 0.03, with both conditions showing curvature away from the post-saccadic distractor location. The reported p-value is Bonferroni-corrected. Based on bins analysis, the statistics suggest that it takes the oculomotor system about 250 ms too update from old to new information about the distractor location.

The primary reason for running Experiment 2 was to determine whether the initial curvature difference in the displacement condition reflects curvature away from the original pre saccadic distractor or whether it reflects curvature towards the new post saccadic distractor. Using Bonferroni-corrected post-hoc pairwise comparisons between the post- saccadic and displacement conditions for each bin showed that the first three bins differed significantly: bin 1,*t*(9) = 5.293, *p* = 0.002, bin 2,*t*(9) = 3.477, *p* = 0.035 and bin 3,*t*(9) = 3.479, *p* = 0.035. In the post-saccadic condition there was curvature away from the distractor location, while in the displacement condition there was curvature away from the pre-saccadic distractor location. The last two bins did not differ significantly between the two conditions.

The results were also smoothed using a Gaussian kernel to extract better temporal precision from the data. The middle panel in Fig. 5B shows curvature difference as a function of time for the three conditions. The results show that the temporal pattern of curvature for the no displacement and displacement conditions are similar to the pattern observed in Experiment 1. The two conditions follow the same pattern until around 160–180 ms, at which point curvature away in the displacement condition switches from curvature away from the original distractor location to curvature away from the displaced distractor location. This again replicates the results from Experiment 1.

To determine whether the time-course in the post-saccadic condition was different from the displacement condition we used the cluster-based permutation described above. There was one cluster with significant differences (*p* < 0.001) between the displacement and post-saccadic condition, from 105 ms to 158 ms. This is exactly when the displacement condition shows curvature away from the original distractor or curvature towards the new distractor. The difference between the displacement and the post-saccadic conditions early in time demonstrates that the curvature reflects curvature away from the original location and not curvature towards the new distractor^25^.

### Combining data from Experiment 1 and Experiment 2

To increase the signal-to-noise ratio and get a better estimate of curvature time-course, the data from the no displacement and displacement conditions in Experiment 1 and 2 were merged, since these conditions were identical in both experiments.

#### Comparison between the no displacement and displacement conditions

**Figure 6:**
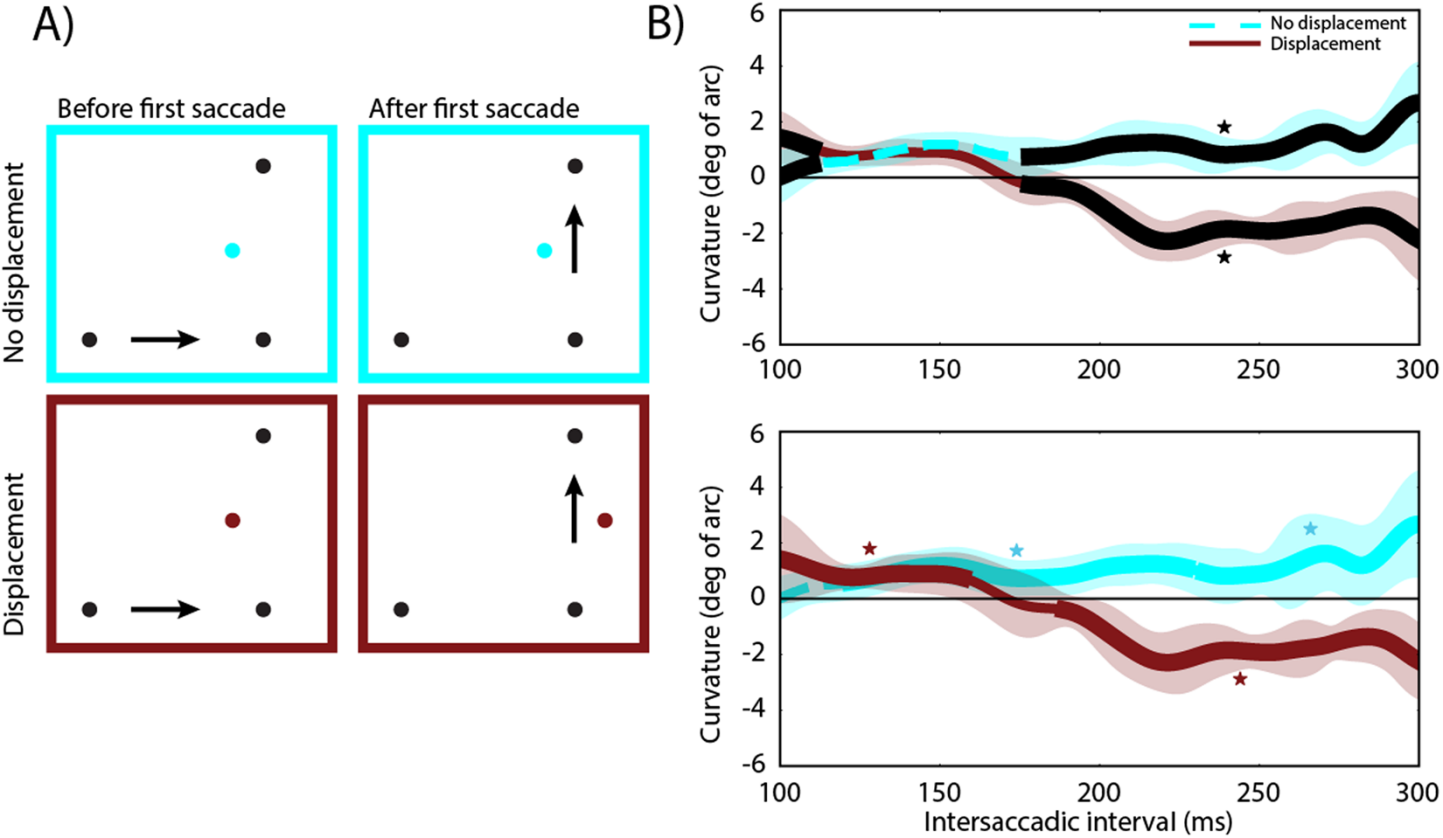
Results for the no displacement and displacement conditions after merging the data from Experiment 1 and 2. (**A**) The position of the distractor before the first saccade and the position of the distractor after the first saccade for each condition. (**B**) Amount of curvature difference (degrees of arc) as a function of intersaccadic interval for each condition. Upper panel: Data smoothed with a Gaussian kernel, with the black line indicating time points were a weighted within-subjects t-test resulted in *p* < 0.05 between conditions. Lower panel: Data smoothed with a Gaussian kernel, with the thick blue line indicating time points were a weighted t-test resulted in *p* < 0.05 from zero for the no displacement condition and with the thick red line indicating time points were a weighted t-test resulted in *p* < 0.05 from zero for the displacement condition. In the no displacement condition positive values indicate curvature away from the distractor. In the displacement condition positive values indicate curvature away from the pre-displacement distractor. The shaded areas are 95% within-subjects confidence intervals. Asterisks indicate significant cluster(s).

A Gaussian kernel was used to smooth the saccade curvature time-course. Cluster-based permutation revealed one non-significant cluster (*p* = 0.45) and one significant cluster (*p* < 0.001) between conditions. The significant cluster started at 175 ms and stayed significant for the remaining time-window (Fig. 6B, upper panel). This suggests that the two conditions showed similar curvature away from the original distractor location for short inter-saccadic intervals and at approximately 175 ms in the displacement condition, saccades started to curvature away from the new displaced distractor location.

#### Saccade curvature time-course separately for the no displacement and displacement conditions

As the main aim of the present study was to investigate the time-course for accommodating new visual information in the oculomotor system across saccades, it is important to determine the saccade curvature time-course separately for each condition. To achieve this, the previously described cluster-based permutation method was adapted for testing against zero. First, the curvature values were demeaned, then the relationship between curvature and time was shuffled, statistics were done against zero to extract cluster sizes and cluster size distributions were build. Ten thousand permutations were done separately for each condition and participant. The results are shown in Fig. 6B (lower panel).

The no displacement condition showed two significant clusters (both clusters, *p* < 0.001), the first one started at approximately 120 ms and lasted until 230 ms, while the second clusters started at approximately 235 ms with sustained curvature away from the distractor for the remaining time window. In the displacement condition there were also two significant clusters (both clusters, *p* < 0.001). There was significant curvature away from the original distractor location from 100 ms until 155 ms, which then shifted to significant curvature away from the new, displaced distractor location at all times greater than 188 ms. This confirms that both conditions showed significant curvature away from the initial distractor location already early in time, while in the displacement condition saccades started to curvature away from the new distractor location somewhere between 155 ms and 188 ms, which is then sustained throughout the remaining time-window.

It is possible that intersaccadic interval was not the only variable affecting the time-course of saccade curvature. For example, the latency of the first saccade could also have an effect. To explore this possibility we computed saccade curvature as a function of the summed latency of the first and second saccade (RT+ISI), see Supplementary Figure 1. As the figure shows, the overall pattern of results is preserved, suggesting that the influence of first saccade RT had to be minimal and that the inter-saccadic interval is responsible for the curvature time-course.

### Discussion

Experiment 2 replicated the same temporal pattern of curvature away for the no displacement and displacement conditions previously found in Experiment 1. Furthermore, saccade curvature time-course in the displacement condition was very different from the post-saccadic condition. Specifically, in the post-saccadic condition, in which the distractor was only presented after the first saccade, saccades never curved towards the distractor, but curved away from the distractor already early in time and this curvature away was sustained over time. These results clearly show that the initial curvature away in the displaced condition cannot be explained by being curvature towards the new distractor^25^, but was in fact initial curvature away from the original distractor location followed by curvature away from the new distractor location.

The results from the merged data pinpointed that the no displacement and displacement condition started to differ significantly at approximately 175 ms. The no displacement conditions showed sustained curvature away starting at about 120 ms. In the displacement condition the discrepancy between the predicted distractor location and the new distractor location was detected at approximately 155 ms. The discrepancy was then resolved over the next 35 ms and after approximately 190 ms the new displaced distractor representation became dominant. The results suggest that the oculomotor system predicts the spatiotopic locations of stimuli across saccades and is able to accommodate new competing visual information in the spatial priority map within 190 ms after a saccade.

## General Discussion

The present results clearly show that the oculomotor system is able to rapidly and automatically update retinal coordinates of behaviorally irrelevant distractor locations, giving rise to spatiotopic representations which replicates previous findings^23^. Here we extend the previous findings by showing that it takes the oculomotor system about 190 ms until new competing visual information becomes dominant on the spatial priority map. When a distractor is displaced during a saccade the oculomotor system is representing the spatial location of the distractor that was available before the start of the saccade sequence up until 190 ms after saccade is completed. This is evident from saccade curvature away from the original distractor location. After 190 ms, saccades start to curve away from the new distractor location. This result clearly illustrates that the oculomotor system is keeping track of changes across saccades. While these distractor displacements are known to typically escape conscious awareness, here we show that the oculomotor system “knew” that a change had taken place.

In addition, Experiment 2 demonstrates that accommodation of new information results in curvature away from the new distractor location at very short intersaccadic intervals. While previous studies have suggested that short latency saccades curve towards distractors and longer latency saccades curve away^25,32,33^, our results indicate that this is not the case for double-step saccades. One possibility for this discrepancy seems to be the fact that reliable information about the second saccade target is available before the start of saccade sequence. This means that updating of the retinal location of the second saccade target biases the competition in its favor. There will be an automatic bias against any new distractor which is presented during the saccade interval, which will lead to a rapid curvature away from its location. Indeed, when in the study by Jonikaitis & Belopolsky^23^ new distractors were presented during the intersaccadic interval they rapidly elicited a massive curvature away. Further corroborating this idea, single saccades do not show curvature towards the distractor when target location is known in advance^34^. Taken together, it appears that rapid updating of the second target location is responsible for curvature away without preceding curvature towards.

Saccade curvature is an implicit measure of the time-course of competition in the oculomotor system. By examining the time-course of saccade curvature we were able to demonstrate that competition in the oculomotor system is driven by the prediction formed before the saccade, up to approximately 190 ms after the saccade is completed. After that, the new information starts to dominate the competition on the spatial priority map in the oculomotor system. For this to occur, the neural representation of the new distractor has to win the competition from the neural representation of the old distractor. While at the earlier intersaccadic intervals both neural representations are most likely to be active at the same time, the 190 ms time point reflects the moment when the new representation dominates the old representation on the oculomotor map. Interestingly, the time-course of updating of the competing representations in the oculomotor system is similar to the one reported in the studies investigating the time-course of shifts of covert spatial attention^35,36^. Given the close relationship between attention and eye movements, there is a possibility that these mechanisms are related^37–39^.

It is widely accepted in the literature that visual changes that occur during saccades or even during screen blanks are hard to detect and often escape conscious perception^1,3,40–42^. The reason for the inability to detect these changes is thought to be the assumption of world stability. When visual features change during these blanks, the visual system attributes these changes to errors in the visual system, as the world is assumed to remain stable and unchanging^1,2,16,17,19,43^. However, these studies all rely on conscious perception of displacement, while perceptual integration can occur in the absence of conscious awareness^41,44^. The current study uses an implicit measure of oculomotor perception which is independent of conscious report. Thus, we are able to separate the representation in the oculomotor system from conscious spatial perception, which might be one of the reasons our results indicate that the oculomotor system is not bound by the assumption of visual stability and does not show saccadic suppression of displacement. Rather it seems that the oculomotor system is predominantly using extra-retinal signals to accurately predict future locations and is therefore able to rapidly detect any mismatches between predictions and new post-saccadic visual information and adjust accordingly. This suggests that while our conscious perception benefits from perceiving the world as stable, it is important for the low-level sensory motor systems to have an accurate, but less stable representation of the world around us. Such mechanism enables us to unconsciously adapt our actions to changes which we do not consciously perceive.

One possible mechanism by which such automatic predictions can be formed is predictive remapping of attended objects ahead of impending eye movement^30,31,45,46^. Indeed, several recent studies have demonstrated that attended locations are rapidly updated across saccades and this is preceded by attentional facilitation at the future retinotopic location of the attended object. This can explain why the oculomotor system is driven by the spatiotopic distractor locations right after the saccade. The present results further demonstrate that the oculomotor system is driven by this prediction up to approximately 190 ms after the saccade.

We should acknowledge that conscious perception of distractor displacement was not explicitly tested in the present study. The main reason for this is that our pilot experiments showed that probing participants awareness on a trial-by-trial basis dramatically interfered with the double-step task, leading to an increase in latencies of both first and second saccades, as well as in a significant increase in error trials (e.g. first saccade landing on the distractor). Therefore, instead of actively probing participants about displacement detection, the displacement size of 1.5° of visual angle was chosen based on change detecting thresholds from previous literature. It has been shown that displacements up to a third of a saccade size go unnoticed, even displacement as large as 4° have been shown to go unnoticed^3,15,47^. In the current experiment the displacement happened during the first saccade (10–12° of visual angle) and thus changes of at least 3° would be expected to go unnoticed, which is well above the 1.5° that the distractor was actually displaced in the current experiment. Additionally, after the experiment participants were asked how many times they saw the distractor move during a block, and verbal reports ranged between 0 and 4 times during a block with 20 displacement trials. Taken together, this strongly suggests that it is unlikely that participants were able to reliably detect the distractor displacement on every trial and if distractor displacements were detected at all, it occurred on a minority of trials.

To conclude, the human eye movement system is remarkably good at keeping track of objects in the outside world. Even slight changes to the locations of irrelevant objects happening during a saccade do not slip past the oculomotor system. The oculomotor system performs rapid and automatic updating of distractor locations, quickly representing them in spatiotopic coordinates after the saccade. About 190 ms after a saccade the oculomotor system has updated its spatial priority map and takes new competing visual information into account. This remarkable automatic location tracking by the oculomotor system enables us to navigate and interact with the world around us without adding the burden of conscious and active updating every time the eyes move.

## Electronic supplementary material

Supplementary information
