# Supplementary material for: Distractor displacements during saccades are reflected in the time-course of saccade curvature
Source: Sci Rep. 2018 Feb 6;8:2469. doi: 10.1038/s41598-018-20578-9 (PMC5802815; doi:10.1038/s41598-018-20578-9)
Supplement: Supplementary file 1 — Supplementary information [file 41598_2018_20578_MOESM1_ESM.pdf]

Distractor displacements during saccades are reflected in the time-course of saccade curvature

Appendix

Jonathan van Leeuwen[1*] & Artem V. Belopolsky[1]

[1]Department of Experimental and Applied Psychology, Vrije Universiteit, Amsterdam, The Netherlands

* Corresponding author:
Email: jvanleeuwen.work@gmail.com

Appendix

Supplementary Table 1

Data rejection and rejection criteria for Experiment 1 and Experiment 2.

	Rejection criteria	Rejected	
		Experiment 1 (11 participants)	Experiment 2 (10 participants)
1	No saccades	0.3%	0.4%
2	Sacc1* RT < 80 ms or > 500 ms	9.6%	14.7%
3	Sacc1* started>2° from fixation dot or landed > 3° from first target dot	7.0%	12.4%
4	Sacc2* started > 2° from the first target dot or ended > 2° from the second target dot	3.4%	3.0%
5	Sacc1* or Sacc2* landed < 2° from distractor dot	4.3%	5.6%
6	Blinked between target onset and Sacc2* landing	0.0%	0.0%
7	Missing samples during one of the saccades	0.3%	0.2%
8	Trial duration > 550ms	6.6%	7.3%
9	Displaced distractor display onset after Sacc1* landing	0.1%	0.0%
10	Sacc1* or Sacc2* speed > 900°/s	0.1%	0.0%
11	Sacc2* curvature deviated > 2.5 std from the mean	1.4%	1.0%
12	Unequal trial numbers between CCW and CW trials	2.2%	1.8%
	Total percentage of trials rejected	35.3%	46.5%

*Sacc1 is the horizontal saccade. Sacc2 is the vertical saccade.

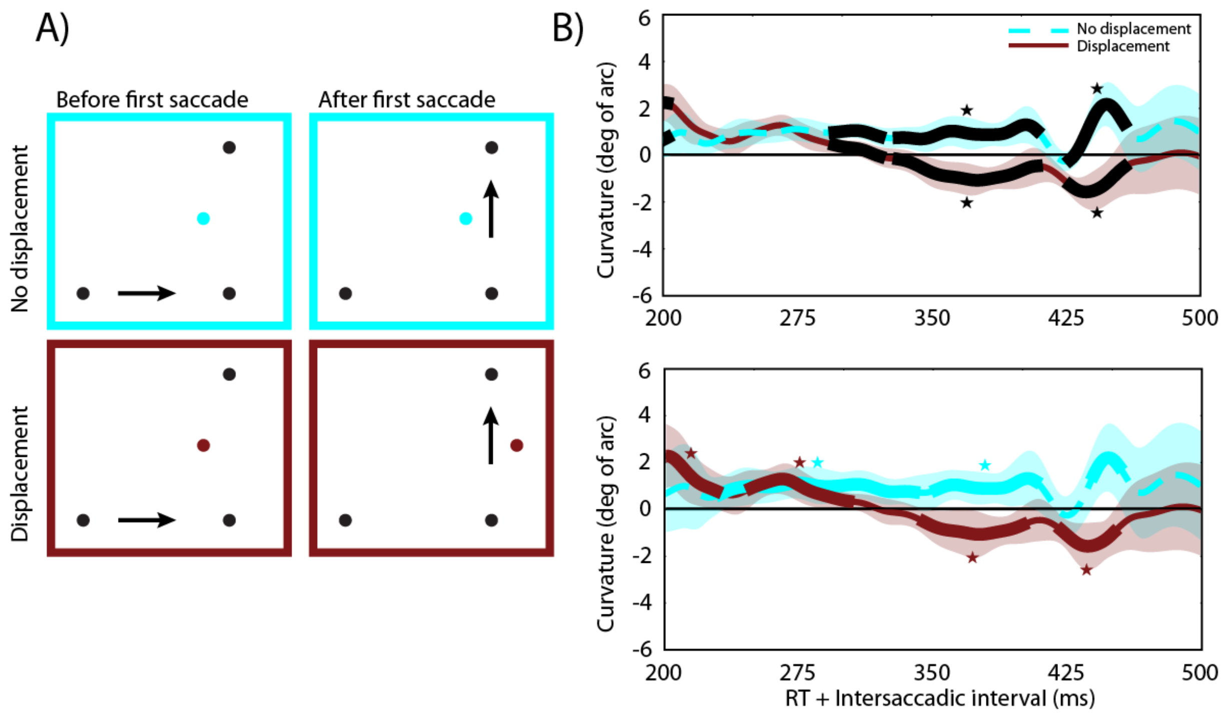

Supplementary Figure 1: The time-course of saccade curvature as a function of summed latency of the first and second saccades (merged data from Experiment 1 and 2). **A**). The position of the distractor before the first saccade and the position of the distractor after the first saccade for each condition. **B**) The curvature difference (degrees of arc) as a function of the latency of the first saccade and the intersaccadic interval for each condition. **Upper panel:** data smoothed with a Gaussian kernel, with the black line indicating time points where a weighted within-subjects t-test resulted in $p<0.05$ between conditions. **Lower panel:** data smoothed with a Gaussian kernel, with the thick blue line indicating time points where a weighted t-test resulted in $p<0.05$ from zero for the no displacement condition and with the thick red line indicating time points were a weighted t-test resulted in $p<0.05$ from zero for the displacement condition. In the no displacement condition positive values indicate curvature away from the distractor. In the displacement condition positive values indicate curvature away from the pre-displacement distractor. The shaded areas are 95% within-subjects confidence intervals. Asterisks indicate significant cluster(s) defined by the permutation testing.
